# Physical Activity Behavior During and After COVID-19 Stay-at-Home Orders—A Longitudinal Study in the Austrian, German, and Italian Alps

**DOI:** 10.3389/fpubh.2022.901763

**Published:** 2022-05-31

**Authors:** Stefanie E. Schöttl, Martin Schnitzer, Laura Savoia, Martin Kopp

**Affiliations:** ^1^Department of Sport Science, University of Innsbruck, Innsbruck, Austria; ^2^Sports Observatory of the Autonomous Province of Bozen/Bolzano—South Tyrol, Bozen, Italy

**Keywords:** COVID-19, physical activity, exercise, longitudinal, European Alps, Austria, Germany, Italy

## Abstract

**Background:**

In Alpine regions, which are very similar due to their topographical location and their wide-ranging sports offerings, the restrictions on sports activities during the COVID-19 pandemic differed in type and level: while in some regions (Tyrol, South Tyrol, Trentino), all sports activities were forbidden except for walking near the home, in other regions (Upper Bavaria, Vorarlberg), people were allowed to go hiking and running during the first lockdown.

**Objective:**

The aim of this study was to investigate the change in physical activity (PA) behavior in different Alpine regions (Upper Bavaria, Vorarlberg, Tyrol, South Tyrol, Trentino) over four periods in 2020, to examine the effects of COVID-19 measures of varying severity on PA behavior and to identify factors associated with a change in PA over time.

**Methods:**

A retrospective online survey was conducted (*N* = 2975) from December 2020, to January 2021. Using the questionnaire of the Eurobarometer 472 study, PA behavior was measured over four periods: before COVID-19 (March), during the first lockdown (March and April), during the relaxed period (May-October) and during the second lockdown (November and December) in 2020.

**Results:**

During the first (M = 5.0h, SD = 4.5) and the second lockdowns (M = 4.9h, SD = 4.3), the participants (age: 42 years, overly active in sports) engaged less in sports than before (M = 5.9h, SD = 4.8) and during the relaxed period in summer (M = 6.4h, SD = 5.0) (average number of hours per week being physically active). A larger percentage of participants from Alpine regions with severe restrictions (Tyrol, South Tyrol, Trentino) decreased their PA during the first lockdown as compared to participants from Upper Bavaria and Vorarlberg with a less strict first lockdown. Those with psychological distress, male participants, and individuals with decreased physical health and less free time during COVID-19 were more likely to reduce their PA.

**Conclusions:**

Despite a short-term negative effect of COVID-19 restrictions on exercise participation during lockdowns, the majority of respondents returned to their original levels of PA during the relaxed COVID-19 phases. As a comparison of Alpine regions shows, particularly severe COVID-19 measures seem to have reduced PA with potential negative health effects. For the future, policy makers and sports organizations should collaborate to support the population in their PA behavior during pandemics to outweigh restrictions.

## Introduction

Since the outbreak of the coronavirus disease (COVID-19), caused by the severe respiratory coronavirus 2 (SARS-CoV-2), in China at the beginning of December 2019, the virus began to spread rapidly around the world (1–3). The emergence of different virus variants (e.g., Delta, Omicron), seasonal changes and environmental factors (e.g., climate, temperature) are responsible for the increase in new COVID-19 cases and new waves of infection worldwide (4–6). Faced with a prolonged situation, the governments of many countries have alternated between strict and relaxed measures to reduce infection rates and to ease pressure on their respective healthcare systems (7, 8). Especially more stringent measures such as the closure of schools, universities and shops, travel restrictions and social distancing clearly have a negative impact on the economy, education, lifestyle and physical and mental health (9–11). In acute phases, governments also restricted opportunities for physical activity (PA). Sports facilities and sports providers (sports clubs, gyms, parks) had to close and a great majority of outdoor PA was prohibited or limited (12, 13).

PA, defined as “any bodily movement produced by skeletal muscles that results in energy expenditure” (14) however, is beneficial for social well-being and physical and mental health (15–23). According to the WHO guidelines, adults (18–64 years) should do at least 150-300 min of moderate-intensity aerobic PA per week, or at least 75–150 min of vigorous-intensity aerobic PA per week, or an equivalent combination of both for substantial health benefits (24). A large study pooling data from population-based surveys showed that in 2016, 27.5% of adults worldwide and 36.8% of adults in high-income Western countries including Austria, Germany and Italy did not comply with these recommendations (25, 26). Physical inactivity and an increase in sitting time in general are a global public health issue (24, 27, 28).

There are concerns that the COVID-19 period would further contribute to people's inactivity because individuals were forced to stay at home and were limited in their freedom to participate in sport (29, 30). In response, several articles have already been published recommending how to stay as active as possible during the COVID-19 pandemic (19, 31–34). The extent to which the COVID-19 period has affected individual PA levels has also been addressed in the literature and is complex (30, 35–37). On the one hand, cross-sectional surveys in many countries showed a short-term decline in PA during the initial lockdown in spring 2020 compared with the pre-COVID-19 period (38–54). On the other hand, participants in some studies also reported increased frequency of PA during the initial stages of the pandemic compared to pre-COVID-19 (39, 53, 55–57). Especially adults who previously engaged in low levels of activity increased their PA during the initial lockdown (48, 58, 59). There are many explanations and approaches as to why individuals did or did not engage in PA during the COVID-19 restrictions (36). Factors associated with a change in PA during lockdown can be gender (42, 48, 55), age (42, 55, 58, 60), ethnicity (61, 62), marital status (60), household composition (63), household income (53, 55, 61, 62), educational level (58), living area (42, 62), access to garden/balcony (61, 63), BMI (53), obesity (61, 63), high-risk medical conditions (53, 63), mental health (53, 63, 64), time (58), screen time (42), closed sport infrastructure (58), availability of home PA equipment (65), dog ownership (65) and prior PA level (61, 66).

It is not clearly established whether there was just a short-term change in PA behavior due to the restrictions or whether people have permanently changed their PA behavior in a positive or negative way (67). Studies investigating the long-term impact of the COVID-19 pandemic on PA behavior provide mixed findings (67). Although most longitudinal studies show a decline in PA levels at the start of the COVID-19 pandemic compared to pre-restrictions (67–70), a variety of changes in PA was observed as restrictions started to ease: The low PA levels of UK participants in the studies of Bu et al. (67) and McCarthy et al. (68) during lockdown either did not or barely increased after restrictions were relaxed. Mata et al. (69), however, observed a steady increase in participants' PA after the initial lockdown in Germany with PA levels in June surpassing pre-lockdown PA. Scottish adults also increased the amount of walking they did after the initial lockdown and returned to pre-lockdown levels (71). While the longitudinal studies that have already been published, primarily examined the changes in PA from pre-lockdown to the period after the initial lockdown, there is little literature that observes a longer time period and includes the subsequent waves of the COVID-19 pandemic (72).

Moreover, studies from different countries and cross-country studies (64, 73–75) examining changes in PA during the pandemic should be taken with caution and can only be partially compared because the severity and the duration of measures varied from country to country and even from region to region (36, 70). To gain a full insight of whether different restrictions may have differing effects on PA behavior (15), it would be beneficial to investigate similar countries or regions.

The following Alpine regions lend themselvesr to comparison because they have very similar sports infrastructures but the restrictions in general and on sports activities differed in type and level during the COVID-19 pandemic: Tyrol (Austria), Vorarlberg (Austria), Upper Bavaria (Bavaria, Germany), South Tyrol (Italy) and Trentino (Italy). The five regions are located wholly or partially in the European Alps in the center of Europe. Their topographical location (nature, mountains,r forests) and the climate offer residents year-round opportunities for nature-based recreation and outdoor activities (76, 77). Furthermore, all regions have wide-ranging sports offerings organized by voluntary, public and private sports sectors: Non-profit sports clubs provide sports and activity programs to all age groups, while state and local governments are responsible for school sports, sports facilities and financing, and the commercial sector is characterized by sports providers such as fitness or health clubs (78). In addition, similarities between the three countries to which the five regions belong, can also be seen in the governance and the healthcare systems (79). Austria and Germany are federal states, South Tyrol and Trentino are autonomous Italian provinces. Therefore, all five regions have extensive decision-making autonomy, which played an important role during the COVID-19 pandemic especially during the initial restrictions (79). Mandatory universal health insurance systems in all three countries guarantee access to primary healthcare for all citizens (1).

In early March 2020, the numbers of COVID-19 cases increased dramatically in Europe, firstly in Italy followed by Austria and Germany (2). Therefore, most European countries progressively implemented restrictions (80). In Italy, the first hard lockdown with severe restrictions started on 08.03.2020, in Austria on 16.03.2020 and in Germany on 23.03.2020 (81). After a decrease in cases in spring, a period of relaxed measures followed in all three countries in summer (7). To hinder the sharply rising pandemic curve in autumn, a new lockdown was implemented in December and November in Austria, Germany and Italy (82). During the first lockdown in March 2020, the measures for citizens in Tyrol, South-Tyrol and Trentino were more restrictive than for people in Upper Bavaria and Vorarlberg. The populations of Tyrol, South Tyrol and Trentino were only allowed to leave their apartments for work, special medical requirements or for necessary groceries (81, 83), while spending time outdoors for jogging, cycling or hiking was not permitted (13). In Upper Bavaria and Vorarlberg, people could also leave their homes for moderate forms of outdoor exercise, while in Upper Bavaria, they could exercise with another member of the same household (81) (overview of restrictions during the pandemic in Table 1).

**Table 1:** Overview of restrictions regarding PA regulations before and during the COVID-19 pandemic in 2020 (simplified presentation of regulations) (84–88).

T1 Before COVID-19	No restrictions
T2 (Mid-)March, April 2020 First lockdown	**In all five regions:** Closed sports facilities, sports clubs, fitness/health centers **Tyrol:** Only walking in the community (19.03-06.04) is allowed, since 07.04: PA outdoors is allowed but no high-risk activities **Vorarlberg:** Walking and running outdoors are allowed **Upper Bavaria:** PA outdoors (no high-risk activities) is allowed **South Tyrol and Trentino:** Only walking near the home is allowed
T3 May-October 2020 Relaxed period	**Gradual opening:** outdoor activities, unorganized PA, organized PA, indoor activities, individual sports, team sports
T4 November, December 2020 Second lockdown	**In all five regions:** Closed sports facilities, sports clubs, fitness/health centers, individual PA outdoors is allowed **Tyrol, Vorarlberg:** Open ski resorts **Upper Bavaria, South Tyrol, Trentino:** Closed ski resorts

To the best of our knowledge, no study to date has assessed the long-term changes in PA behavior due to different restrictions during the COVID-19 pandemic in similar regions. The aim of our study is 1) to investigate the longitudinal change in PA behavior in different Alpine regions over four periods in 2020, 2) to examine the effects of COVID-19 measures of varying severity on PA behavior, 3) and to identify factors associated with a change in PA over time.

## Materials and Methods

### Study Design and Data Collection

A retrospective online survey was conducted from December 12, 2020, to January 31, 2021 to investigate changes in PA, health and lifestyle behaviors due to COVID-19 restrictions. The online questionnaire (available on request from the authors) was distributed and promoted *via* newspapers, radio, social media and websites of various partners. For data collection, the online survey software “SoSci Survey” was used (https://www.soscisurvey.de/). To ensure comprehensibility and readability of the questionnaire and to increase response rates (89), a pretest was completed and the questionnaire was subsequently adapted. Individuals who were 18 years or older and who stayed at least temporarily in one of the five regions during the COVID-19 pandemic (Tyrol, South Tyrol, Trentino, Upper Bavaria and Vorarlberg) were invited to participate in the online survey. The questionnaire was provided in two languages, German and Italian. Before filling out the questionnaire, the participants received information about the aim of the survey, the inclusion criteria, the duration of the questionnaire and privacy and data protection. At the beginning of the questionnaire, a table reminded participants of the COVID-19 measures taken in the four different periods (see Table 1). Participants took an average of 17 ± 9 min to complete the questionnaire. The survey was conducted according to the “ethical guidelines for surveys” approved by the Institutional Review Board (IRB) of the Department of Sport Science as well as the Board for Ethical Issues (BfEI) of the University of Innsbruck.

### Measures

We compiled a self-administered questionnaire consisting of four parts: 1) sociodemographic data, 2) mental health status and lifestyle behavior, 3) PA behavior, 4) COVID-19 pandemic. Participants could also complete an optional fifth part about sports events. The optional part is not discussed in more detail because it was not included in this study.

#### Sociodemographic Data

Sociodemographic information was collected to describe and compare the participants of the five regions and to determine potential predictors and covariates (90–92) influencing PA behavior during the COVID-19 pandemic. This part of the questionnaire included gender (male, female, diverse), age, body mass index (BMI), education, personal monthly income, employment status, home-office status, marital status, migration background, household size, household composition (living alone, living with others but no children, living with children including others), private access to garden/terrace/balcony and regional affiliation.

#### Mental Health Status and Lifestyle Behavior

The K6 screening scale (K6 scale) (93, 94) was used to measure non-specific psychological distress in participants. Respondents determined how they have been feeling during the past 30 days (6 items) on a 5-point Likert scale. After scoring, individuals were classified in two groups: scores of 13–24 indicate probable SMI (severe mental illness), whereas scores of 0–12 do not (95).

In addition, participants were asked how various health and lifestyle behaviors had changed since the onset of the COVID-19 pandemic and the restrictions. Change in self-rated “quality of life”, “physical health,” “mental health,” “income,” “sleep quality” and “mood” were assessed using a 5-item Likert scale (much worse, somewhat worse, stayed the same, somewhat better, much better). The change in further health and lifestyle behaviors (“leisure time,” “time for yourself,” “time with family,” “time with friends,” “time in nature/outdoors,” “sleep duration,” “stress,” “boredom,” “loneliness”) were rated using a 5-point Likert scale (much lower, lower, about the same, higher, much higher). Changes in “screen time,” “social media use,” “participation in e-sports,” “gardening/do-it-yourself (DIY),” “healthy food,” “unhealthy food,” “smoking” and “alcohol consumption” were evaluated using the same Likert scale and participants could also select the option “not applicable.”

#### Physical Activity Behavior

Questions on PA behavior were partly taken or adapted from the questionnaire of the Eurobarometer 472 study (96) and supplemented with specific questions on PA during COVID-19. The frequency of PA (never, seldom, 1–3 times a month, 1–2 times a week, 3–4 times a week, 5 times a week or more), amount of PA (average number of hours per week), type of PA (the 3 most frequently practiced sports), locations of PA (in a park/outdoors etc., on the way between home and school, work or shops, at home, at work, at school or university, at a sports center, at a sports club, at a health or fitness center) and with whom (alone, with partner/children/family, with friends, under guidance/trainer, in a training group, with DVDs/videos/online tools/TV programs) were reported for four different time points: pre COVID-19, during the first lockdown, during the relaxed period, during the second lockdown. Participants were also asked whether they are members of clubs, where they participate in sport (sports club, health or fitness center, other, not a member of any club), whether they engage in PA regularly and why, how they rate the sports infrastructure in their region, whether they used new sports offerings during the COVID-19 restrictions and whether it was easy for them to stay physically active during the COVID-19 pandemic.

#### COVID-19 Pandemic

The fourth section of the questionnaire asked respondents about their personal experiences with COVID-19 and their opinion about the COVID-19 restrictions. This included the following questions: “Have you tested positive for COVID-19?” “Has anyone in your immediate environment tested positive for COVID-19?” Have you already been in quarantine?” “Do you belong to the COVID-19 risk group?” “Are you worried about the COVID-19 pandemic?” “How satisfied are you with 1) the COVID crisis management of your national government 2) the COVID crisis management of your regional government 3) the regulations your government has taken regarding PA during the COVID-19 pandemic?” (not at all satisfied, slightly satisfied, satisfied, very satisfied).

### Data Analysis

For the descriptive presentation of the data, number and percent are reported for categorial (nominal or ordinal) variables and mean (M) and standard deviation (SD) for continuous variables. To evaluate significant differences between sociodemographic and PA-related variables and the five regions, an χ^2^-test (categorial variables) and one-way ANOVA (continuous variables: age, BMI, household size) were conducted. If no homogeneity of variance was asserted with Levene's Test (*p* < 0.05), the Welch-ANOVA was interpreted (97). To determine significant differences in the amount of PA (weekly average numbers of hours per week) over four time periods in the five different regions, a repeated measures ANOVA and Bonferroni-adjusted *post-hoc* analysis for pairwise comparisons were performed. For violations of sphericity (*p* < 0.05) verified by Mauchly's Test, the Greenhouse-Geisser adjustment was used. To determine the change of frequency of PA and if participants increased, decreased or did not change their frequency of PA, the difference in PA levels between T1 vs. T2, T3 vs. T4, and T1 vs. T2+T3+T4 was calculated. Afterwards, the subjects were assigned to the corresponding groups (increased, decreased, no change). A multiple linear regression was performed to predict changes in PA behavior (continuous PA variable: T1 vs. T2+T3+T4 before group assignment) with several predictor variables. IBM SPSS Statistics (version 26) was used for data analysis and statistical significance was declared if *p* < 0.05.

## Results

### Participants

After data cleaning, a total of 2,975 participants were included in the study. Except for Upper Bavaria (*n* = 467), the number of included participants exceeded 500 in every region. Among all participants, there were more women (53%) than men. On average, the participants were 42 years old, had a BMI (kg/m^2^) of 24 ± 3.8 and a high education level. Over one-third of the respondents were married or in a relationship. Only a small proportion of participants lived alone (14.1%), with an average of 2.8 persons per household. Over 90% of all participants had private access to a garden/terrace or balcony. The outcome of the K6 scale showed that 11.4% of all participants could be classified as probably having SMI. At the time of data collection in December 2020 and January 2021, 10% of the participants had tested positive for COVID-19 and 9.1% belonged to the COVID-19 risk group. 55% of the sample were members of sports clubs and 17% of fitness or health centers. 88% of all participants indicated that they engaged in PA regularly. Detailed demographic and PA-related characteristics of participants of the five Alpine regions can be seen in Table 2. Despite significant differences between the demographic and PA-related variables and the variable “region,” the characteristics of participants from the five Alpine regions were very similar and can be compared.

**Table 2:** Demographic and PA-related characteristics of participants of five Alpine regions.

**Variable *N* (%) or mean ±SD**	**Tyrol (TY) (*n* = 611)**	**Vorarlberg (VO) (*n* = 696)**	**Upper Bavaria (UB) (*n* = 467)**	**South Tyrol (ST) (*n* = 576)**	**Trentino (TN) (*n* = 625)**	**Total (*n* = 2975)**	***p*-value**
Gender							<0.001
Female	300 (49.1)	382 (54.9)	297 (63.6)	305 (53.0)	278 (44.5)	1562 (52.5)	
Male	310 (50.7)	312 (44.8)	167 (35.8)	269 (46.7)	347 (55.5)	1405 (47.2)	
Other	1 (0.2)	2 (0.3)	3 (0.6)	2 (0.3)	-	8 (0.3)	
Age (years)	39.5 ± 15.3	42.2 ± 12.6	42 ± 14.2	43.9 ± 14.2	43.7 ± 13.5	42.3 ± 14.0	<0.001^**a**^
BMI (kg/m^2^)	24.1 ± 4.0	24.3 ± 3.9	24.4 ± 4.5	23.1 ± 3.0	23.6 ± 3.7	23.9 ± 3.8	<0.001^**b**^
Marital status							0.005
Single	194 (31.8)	164 (23.6)	151 (32.3)	169 (29.3)	188 (30.1)	866 (29.1)	
Partner/married	417 (68.2)	532 (76.4)	316 (67.7)	407 (70.7)	437 (69.9)	2109 (70.9)	
Education							
Low	36 (5.9)	24 (3.4)	93 (19.9)	65 (11.3)	51 (8.2)	269 (9.0)	<0.001
Middle	288 (47.1)	380 (54.6)	160 (34.3)	306 (53.1)	307 (49.1)	1441 (48.4)	
High	287 (47.0)	292 (42)	214 (45.8)	205 (35.6)	267 (42.7)	1265 (42.5)	
Personal monthly income (net)							<0.001
< € 1000	148 (24.2)	46 (6.6)	81 (17.3)	101 (17.5)	138 (22.1)	514 (17.3)	
€1000 - < €2000	171 (28.0)	180 (25.9)	109 (23.3)	238 (41.3)	282 (45.1)	980 (32.9)	
€2000 - <	234 (38.3)	390 (56.0)	216 (46.3)	190 (33.0)	138 (22.1)	1168 (39.3)	
No response	58 (9.5)	80 (11.5)	61 (13.1)	47 (8.2)	67 (10.7)	313 (10.5)	
Household size	2.7 ± 1.2	2.8 ± 1.2	2.5 + 1.2	3.0 ± 1.3	3.1 ± 1.3	2.8 + 1.3	<0.001^**c**^
Household composition							<0.001
Living alone	77 (12.6)	95 (13.6)	103 (22.1)	74 (12.8)	71 (11.4)	418 (14.1)	
Living with others but no children	355 (58.1)	310 (44.5)	218 (46.7)	240 (41.7)	237 (37.9)	1356 (45.6)	
Living with children (including others)	179 (29.3)	291 (41.8)	146 (31.3)	262 (45.5)	317 (50.7)	1195 (40.2)	
Private access to garden/terrace/balcony							<0.001
No	66 (10.8)	20 (2.9)	40 (8.6)	24 (4.2)	37 (5.9)	187 (6.3)	
Yes	545 (89.2)	676 (97.1)	427 (91.4)	552 (95.8)	588 (94.1)	2788 (93.7)	
K6 Scale (*N* = 2945)							<0.001
No SMI	544 (90.1)	636 (92)	403 (88)	515 (90)	511 (82.4)	2609 (88.6)	
SMI	60 (9.9)	55 (8)	55 (12)	57 (10)	109 (17.6)	336 (11.4)	
COVID-19 positive (*N* = 2711)							<0.001
No	503 (91.3)	582 (91.5)	400 (94.3)	460 (87.5)	491 (85.5)	2436 (89.9)	
Yes	48 (8.7)	54 (8.5)	24 (5.7)	66 (12.5)	83 (14.5)	275 (10.1)	
COVID-19 risk group (*N* = 2702)							0.001
No	490 (89.1)	580 (91.6)	365 (86.3)	484 (92.5)	536 (93.5)	2455 (90.9)	
Yes	60 (10.9)	53 (8.4)	58 (13.7)	39 (7.5)	37 (6.5)	247 (9.1)	
Members of sports club	370 (60.9)	327 (47.8)	214 (46.5)	279 (49.5)	421 (68.8)	1611 (55.0)	<0.001
Members of fitness/health center	101 (16.6)	137 (20.0)	107 (23.3)	80 (14.2)	67 (10.9)	492 (16.8)	<0.001
Regular PA (at least once a week)	564 (92.9)	618 (89.3)	404 (87.6)	505 (88.6)	503 (81.9)	2594 (88.1)	<0.001

*χ^2^ tests and one-way ANOVA (age, BMI, household size) were conducted to evaluate significance of differences in characteristics of five participant groups; post-hoc tests showed significant differences between the following regions; ^a^TY and VO, TY and UB, TY and ST, TY and TN; ^b^ST and TY, ST and VO, ST and UB, TN and VO, TN and UB; ^c^ST and TY, ST and VO, ST and UB, TN and TY, TN and VO, TN and UB, VO and UB*.

### Amount of Physical Activity

The mean and standard deviation of the amount of PA (average number of hours per week) over four time periods (T1–T4) is shown in Table 3. Bonferroni-adjusted *post-hoc* analysis reported significant differences for the amount of PA for participants from Tyrol, South Tyrol and Trentino between T1 and T2 but not for participants from Vorarlberg and Upper Bavaria, who were subject to a less stringent first lockdown. The pairwise comparison of the amount of PA between T3 and T4 showed significant differences for all Alpine regions. During the easing of restrictions in summer, participants from all regions not only returned to pre-lockdown PA levels, but also surpassed the pre-lockdown level with no significant differences between T1 vs. T3 except for Vorarlberg (*p* < 0.05). While individuals from Tyrol, South Tyrol and Trentino invested a similar average number of hours per week in PA during both lockdowns in spring and winter 2020 (no significant differences between T2 and T4), the PA levels of people in Vorarlberg and Upper Bavaria were significantly lower in the second lockdown (T4) than in the first lockdown (T2).

**Table 3:** Amount of PA in the five Alpine regions at four time points.

**Mean ±SD**	**T1**	**T2**	**T3**	**T4**	**T1 vs. T2**	**T2 vs. T3**	**T3 vs. T4**	**T1 vs. T3**	**T2 vs. T4**
Tyrol (*n* = 219)	6.8 ± 5.4	4.9 ± 4.6	7.1 ± 5.6	5.3 ± 4.8	<0.001	<0.001	<0.001	0.344	0.627
Vorarlberg (*n* = 296)	4.9 ± 3.8	4.8 ± 4.1	5.5 ± 4.3	4.4 ± 3.6	1.000	<0.001	<0.001	0.001	0.003
Upper Bavaria (*n* = 162)	6.7 ± 5.5	6.3 ± 5.6	7.0 ± 5.6	5.3 ± 4.9	1.000	0.102	<0.001	1.000	<0.001
South Tyrol (*n* = 174)	5.8 ± 4.6	4.5 ± 3.7	6.3 ± 5.1	5.1 ± 4.3	<0.001	<0.001	<0.001	0.107	0.133
Trentino (*n* = 45)	5.4 ± 4.1	3.8 ± 3.7	5.8 ± 3.9	4.0 ± 3.3	0.003	<0.001	<0.001	1.000	1.000
Total (*n* = 896)	5.9 ± 4.8	5.0 ± 4.5	6.4 ± 5.0	4.9 ± 4.3	<0.001	<0.001	<0.001	<0.001	1.000

*Mean ± SD and results of one-way repeated-measures ANOVA and post-hoc tests of amount of PA (average number of hours per week) in the five Alpine regions at four time points (T1–T4)*.

### Frequency of Physical Activity

**Figure 1:**
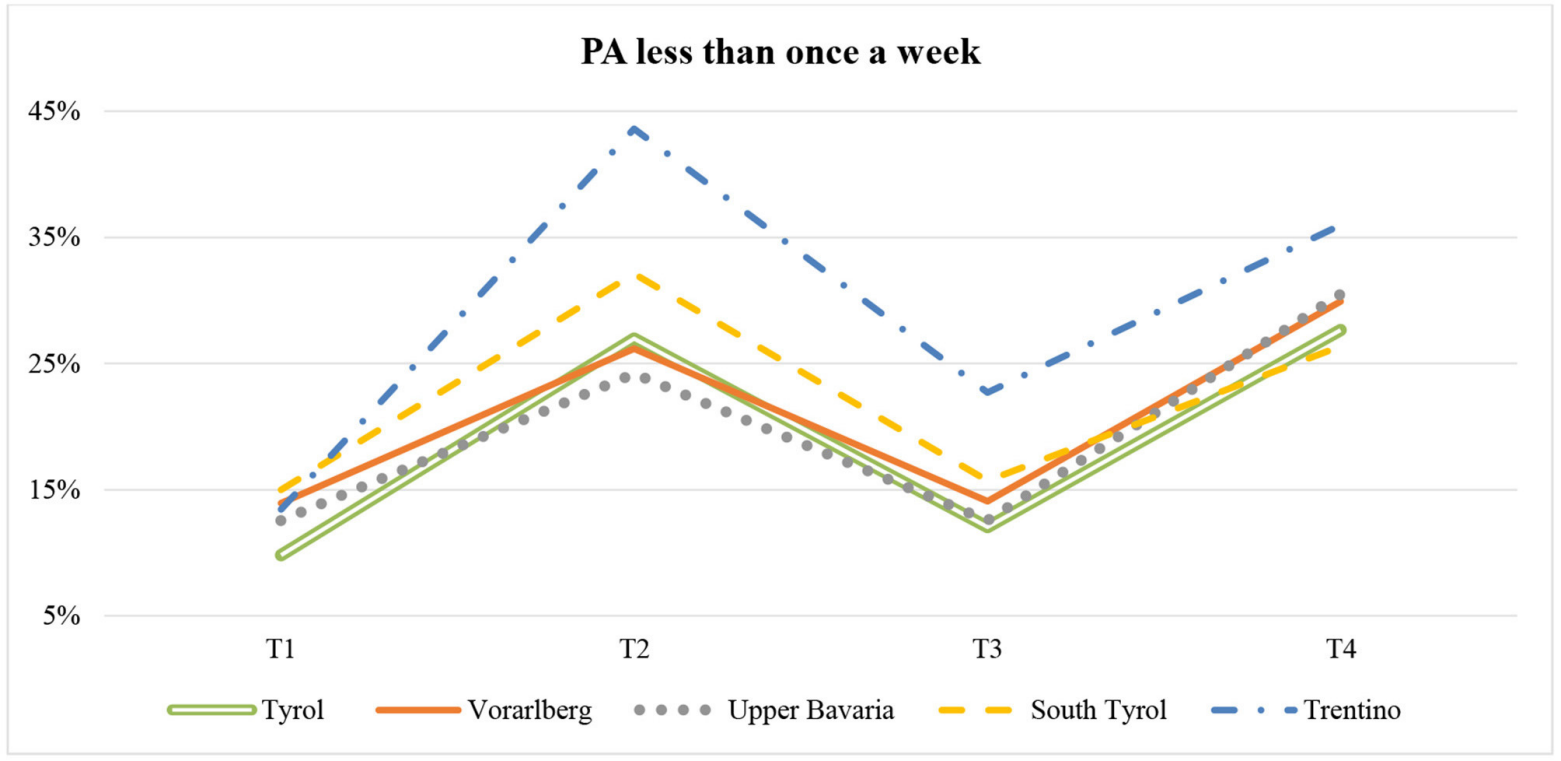
Frequency of PA (PA less than once a week) over four time points (T1-T4) in five Alpine regions (Tyrol: *n* = 582; Vorarlberg: *n* = 668; Upper Bavaria: *n* = 446; South Tyrol: *n* = 554; Trentino: *n* = 603).

Participants from the five Alpine regions reported the frequency of PA over four time periods (T1-T4). The Supplementary Table 1 shows how often participants from the five regions engaged in PA at the different time points. For a simplified representation of the data, the PA levels were grouped together under “PA less than once a week” (never, seldom, 1–3 times a month) and “PA at least once a week” (1–2 times a week, 3–4 times a week, 5 times a week or more). At T1, only 10% to 15% of the participants from the five regions exercised less than once a week (see Figure 1). The second period (T2) led to a noticeable increase in people engaging in PA less than once a week especially in regions with more restrictive measures (Trentino: 43.6%, South Tyrol: 32.1%) in contrast to the regions Upper Bavaria (24.2%) and Vorarlberg (26.2%). While the number of people exercising less than once a week, decreased during the relaxed phase (T3), this number increased again in the fourth period (T4). Participants from Trentino were also less physically active at T3 and T4 than those from the other Alpine regions. The χ^2^-test showed no significant difference between frequency of PA and regional affiliation at T1 (*p* = 0.95) but it did at T2, T3 and T4 (*p* < 0.05).

### Change of Frequency of Physical Activity

During the first lockdown (T2) compared with the pre-COVID-19 period (T1), a total of 42.5% of all participants reduced their frequency of PA. 20.3% of all participants increased their level of PA and 37.3% did not change their PA level (see Table 4). In Alpine regions with stricter measures during the first lockdown like Tyrol (43.3%), South Tyrol (43.7%) and Trentino (57.5%), the proportion of those who reduced their PA was higher than in regions with less strict measures like Vorarlberg (32.6%) and Upper Bavaria (34.3%). The comparison of the change in PA during the November/December lockdown (T4) vs. the relaxed period in summer 2020 (T3) showed that only 8.3% of all participants increased their frequency of PA, with 52.9% not changing their PA levels and 38.8 decreasing their PA levels. There were no clear or significant differences between the Alpine regions in terms of the change in PA during T3 vs. T4. The change in PA over time, and during all three time periods during COVID-19 (T2, T3, T4) compared with pre-COVID-19, demonstrated a higher decrease in PA in Alpine regions with stricter measures (Tyrol, South Tyrol, Trentino) than in regions with less strict measures (Vorarlberg, Upper Bavaria).

**Table 4:** Change of frequency of PA in five Alpine regions.

**N (%)**	**T1 vs. T2**			**T3 vs. T4**			**(T1 vs. T2, T3, T4)**		
	**(*****p*** **<** **0.001)**			**(*****p*** **=** **0.77)**			**(*****p*** **<** **0.001)**		
	**Increase**	**No change**	**Decrease**	**Increase**	**No change**	**Decrease**	**Increase**	**No change**	**Decrease**
Tyrol (*n* = 582)	116 (19.9)	214 (36.8)	252 (43.3)	57 (9.8)	291 (50.0)	234 (40.2)	122 (21.0)	171 (29.4)	289 (49.7)
Vorarlberg (*n* = 668)	154 (23.1)	296 (44.3)	218 (32.6)	51 (7.6)	366 (54.8)	251 (37.6)	192 (28.7)	213 (31.9)	263 (39.4)
Upper Bavaria (*n* = 446)	101 (22.6)	192 (43.0)	153 (34.3)	29 (6.5)	220 (49.3)	197 (44.2)	113 (25.3)	141 (31.6)	192 (43.0)
South Tyrol (*n* = 554)	116 (20.9)	196 (35.4)	242 (43.7)	51 (9.2)	311 (56.1)	192 (34.7)	135 (24.4)	153 (27.6)	266 (48.0)
Trentino (*n* = 603)	91 (15.1)	165 (27.4)	347 (57.5)	48 (8.0)	321 (53.2)	234 (38.8)	97 (16.1)	133 (22.1)	373 (61.9)
Total (*n* = 2853)	578 (20.3)	1063 (37.3)	1212 (42.5)	236 (8.3)	1509 (52.9)	1108 (38.8)	659 (23.1)	811 (28.4)	1383 (48.5)

*Number and percentage of participants in five Alpine regions who increased, decreased or did not change their frequency of PA during T1 vs. T2, during T3 vs. T4 and over time T1 vs. T2+T3+T4) (χ^2^ tests were conducted to evaluate the significant differences between “change in PA” and “region”)*.

### Predictors of PA Increase

The multiple linear regression model was statistically significant, F _(23, 25, 11)_ = 25.49, *p* < 0.001. The R^2^ for the overall model was 0.19 (adjusted R^2^ = 0.18) and showed a medium goodness-of-fit (98). Sixteen variables were entered into the regression model. Eight variables showed no significant effect (BMI, education, marital status, household composition, COVID-19 positive, COVID-19 risk group, mental health, stress during COVID-19), while six variables and two categories of two variables contributed significantly to predicting the change in PA during the COVID-19 crisis. If the age of the participants decreased by 1 year, the PA over time increased by 0.004. Private access to a garden/terrace/balcony, working from home during the crisis and increased physical health during COVID-19 contributed to an increase in PA during COVID-19. For male participants, individuals with SMI, for participants whose physical health decreased during the crisis and for people with less free time during COVID-19, the PA over time decreased compared to the reference categories. Decreased (β = −0.212) and increased physical health (0.205) during COVID-19 had the greatest influence on the change in PA over time, while age (β = −0.045) was the factor with the least influence. Detailed values for linear regression can be found in Table 5.

**Table 5:** Associations between increased PA behavior and several variables.

**Variable**	**Category**	**Increase of PA over COVID-19 time**		
		**Unstandardized b**	**Standardized β**	**Sig**.
Gender	Female (Reference)			
	Male	−0.198	−0.093	<0.001*
Age		−0.004	−0.045	0.038*
BMI		−0.007	−0.024	0.205
Education	Low (Reference)			
	Middle High	0.035 0.075	0.016 0.035	0.622 0.307
Marital status	Single (Reference)	0.021	0.009	0.687
	Partner/married			
Household composition	Living alone (Reference)			
	Living with others but no children Living with children (including others)	−0.082 −0.097	−0,038 −0.045	0.217 0.168
Private access to garden/terrace/balcony	No (Reference)			
	Yes	0.259	0.057	0.002*
Working from home	No (Reference)			
	Yes/Part-time	0.180	0.080	<0.001*
K6 Scale	No SMI (Reference)			
	SMI	−0.183	−0.054	0.006*
COVID-19 positive	No (Reference)			
	Yes	−0.104	−0.029	0.111
COVID-19 risk group	No (Reference)			
	Yes	0.125	0.032	0.090
Quality of life	Same during COVID-19 (Reference)			
	Decreased Increased	−0.017 −0.170	−0.008 −0.049	0.724 0.030*
Physical health	Same during COVID-19 (Reference)			
	Decreased Increased	−0.491 0.577	−0.212 0.205	<0.001* <0.001*
Mental health	Same during COVID-19 (Reference)			
	Decreased Increased	−0.066 0.099	−0.031 0.024	0.163 0.261
Free time	Same during COVID-19 (Reference)			
	Less More	−0.264 -0.017	-0.107 −0.008	<0.001* 0.718
Stress	Same during COVID-19 (Reference)			
	Less More	0.059 0.015	0.023 0.007	0.284 0.756

*Results of the multiple linear regression analysis examining associations between change in PA behavior during the COVID-19 crisis and several variables (n = 2535). The bold values indicate the statistically significant p-values which are <0.05.*

### Characteristics of PA Behavior

**Figure 2:**
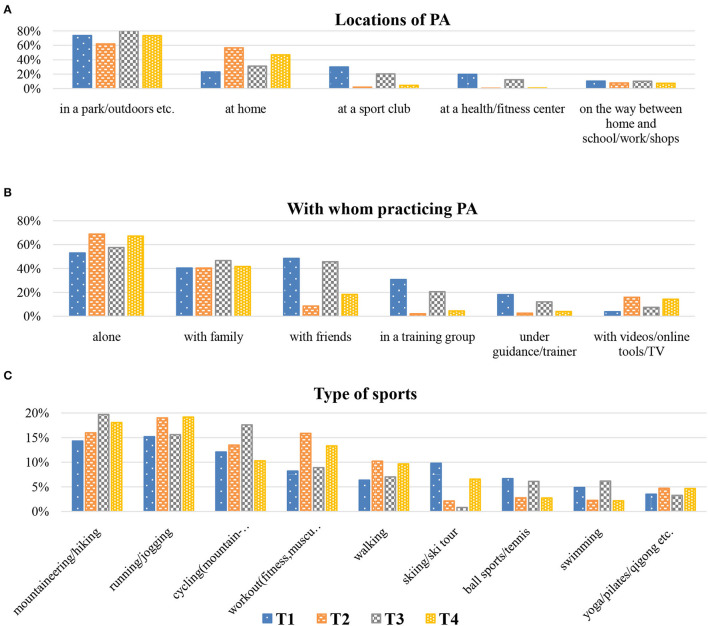
**(A)** Locations of PA, **(B)** with whom participants practiced PA and **(C)** type of sports, over four time periods (T1–T4) for the total sample.

Figure 2 presents where (a) and with whom participants practiced PA over four time points (T1–T4) (b) and in which type of sports they took part (c). This figure summarizes the results of all participants from the five regions. Detailed numbers of participants from the five Alpine regions can be found in Supplementary Table 2. Over all periods (T1–T4), participants were consistently physically active in parks/outdoors with higher outdoor activity in periods either without or with relaxed restrictions. During strict measures (T2, T4), individuals did more PA at home and little to no PA at sports clubs or health/fitness studios. In addition, exercising with friends, in a training group or under guidance/with a trainer were also reduced during strict phases, whereas the number of people doing sport alone or with videos/online tools/TV increased. Over all periods, the participants of our study primarily preferred outdoor sports. In addition to running/jogging and walking, the number of individuals who performed workouts (fitness, muscle strength) and yoga/pilates/qigong also increased during periods with strict measures.

About 40% of participants from Tyrol, Vorarlberg, Upper Bavaria and South Tyrol and 57% of participants from Trentino reported that it was not easy for them to stay physically active during the COVID-19 pandemic. The main reasons given by the participants were fewer or no opportunities, less or no motivation and less or no time during COVID-19 restrictions.

However, between 25% (Trentino) and 40% (Upper Bavaria) of participants (Vorarlberg: 28%, Tyrol: 37%, South Tyrol: 31%) used new sports offerings/formats during the COVID-19 pandemic. Online offers for PA, new or alternative types of sports and home training were popular during the crisis.

### Satisfaction With COVID Crisis Management

**Figure 3:**
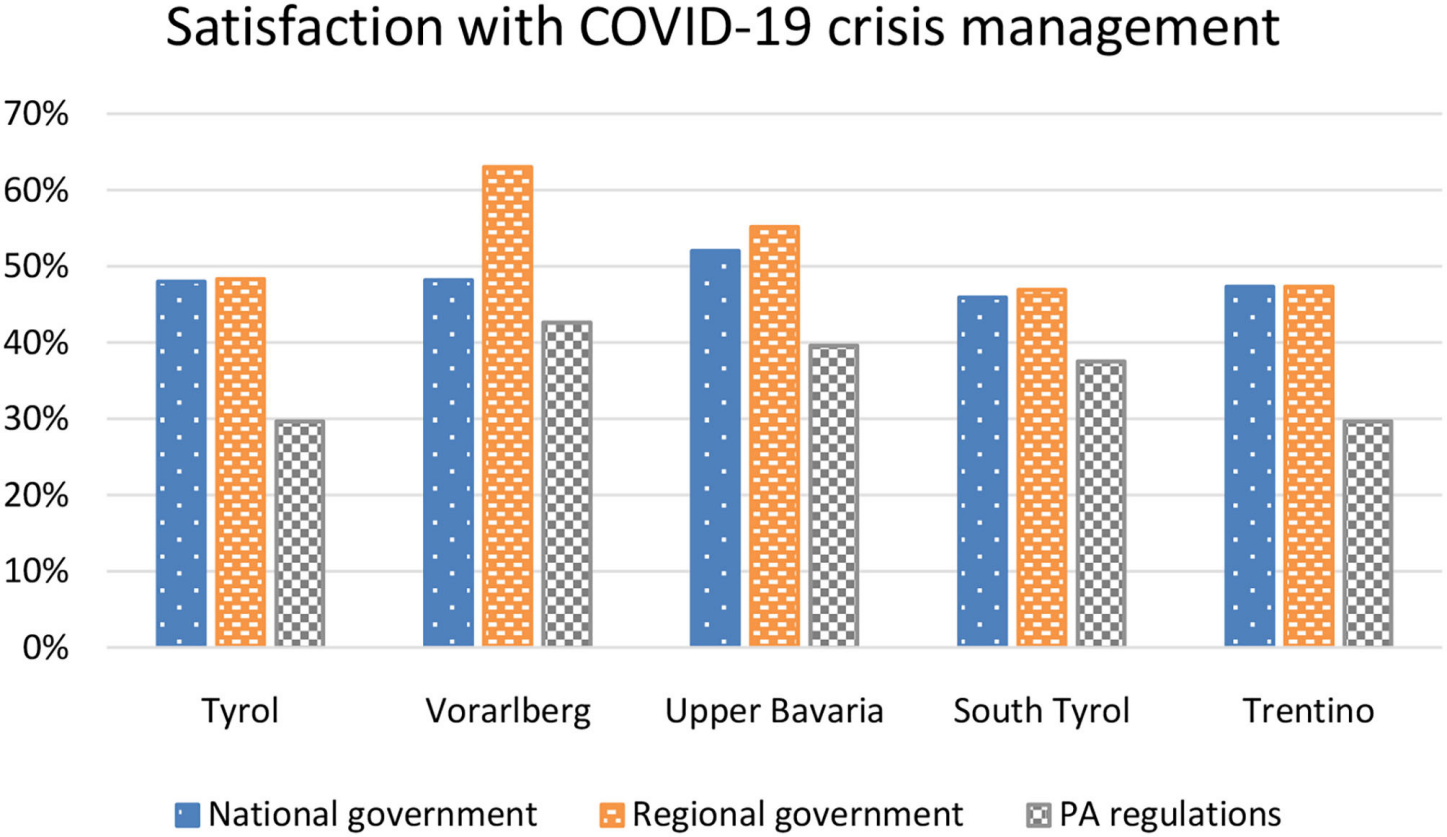
Satisfaction of participants from the five Alpine regions with the COVID-19 crisis management of their national governments, their regional governments and the PA regulations their governments had taken.

Participants were asked how satisfied (not at all satisfied, slightly satisfied, satisfied, very satisfied) they were with the COVID crisis management of their national governments, their regional governments and the regulations their respective governments had taken regarding PA during the COVID-19 pandemic. For a simplified representation of the data, satisfaction was grouped together under satisfied” (satisfied, very satisfied) and “unsatisfied” (not at all satisfied, slightly satisfied). As can be seen in Figure 3, people from Vorarlberg and Upper Bavaria, regions with less strict measures, were more satisfied with the COVID-19 crisis management than individuals from Tyrol, South Tyrol and Trentino. The proportion of participants satisfied with their national government's crisis management was the same in Tyrol and Vorarlberg (48%), two Austrian states. However, participants from Vorarlberg, whose regional government followed a softer course, especially in the first lockdown in 2020, were considerably more satisfied with their regional government (63%) and with the PA regulations (43%) of their regional government than the Tyrolean participants.

## Discussion

In a large sample from five different Alpine regions, we investigated the longitudinal change in sports behavior over four periods in 2020 and examined the effects of COVID-19 measures of varying severity on PA behavior. Factors associated with changes in PA over the four periods were also identified.

Results relating to the amount of PA (average number of hours per week) and frequency of PA (how often participants engaged in PA) showed a decrease in PA levels during the first COVID-19 measures compared to pre-restrictions in all five Alpine regions. While participants returned to pre-lockdown PA levels during the easing of restrictions in summer 2020, PA levels declined again in the second lockdown in November and December 2020. Moreover, differences in participants' PA over four time periods were evident in regions with less stringent measures vs. regions with more stringent measures. Particularly during the first lockdown, individuals from Tyrol, South Tyrol and Trentino were less physically active and a larger proportion of individuals reduced their PA compared to individuals from Vorarlberg and Upper Bavaria, with less stringent PA restrictions. In addition to a reduction in PA during the lockdowns, an increase in PA was also observed. While 20% of all participants increased their PA during the first lockdown compared to pre-lockdown, only 8% were more physically active during the second lockdown compared to the previous relaxed summer period. Factors that were positively associated with an increase in PA during the COVID-19 crisis were private access to a garden/terrace/balcony, working from home and increased physical health during the pandemic. Those with SMI, male participants, and participants with decreased physical health and less free time during COVID-19 were more likely to reduce their PA.

The present findings in our study are similar to those of previous surveys from Austria, Germany and Italy which also reported a short-term decline in PA during the initial lockdown in spring 2020. Haider et al. (40) reported a 13% decrease in moderate-to-vigorous PA during the initial lockdown compared to pre-COVID-19 in Austrian people with a mean age of 36 years. Schlichtiger et al. (44) also observed an overall PA decrease during the first lockdown in older Bavarian (German) adults. In the study by Maugeri et al. (48), Italian adults were significantly less physically active during the COVID-19 emergency compared to the period before restrictions. However, in addition to a decrease in PA, studies from Tyrol (Austria) (39), Germany (43) and Italy (57) also observed increased PA levels during the initial stage of the pandemic which was in line with our findings especially during the first lockdown in 2020.

Longitudinal studies investigating changes in PA over a longer period provided different results compared to our study. In two studies in the UK (67, 68), people were unable or less able to increase their low lockdown PA levels during the relaxed summer period. On the other hand, in two surveys with Scottish (71) and German (69) adults, participants returned to pre-lockdown PA levels during the relaxed period or even surpassed pre-lockdown PA levels (69). These findings are consistent with our findings. To the best of our knowledge, there are not yet any studies examining long-term changes in PA during COVID-19 in Austria and Italy. Furthermore, there is currently little literature observing changes in PA behavior over a longer period including subsequent waves of COVID-19 restrictions. Rogers et al. (99) analyzed data from April and November 2020 to survey how COVID-19 affected exercise patterns in American adults. In November, the frequency of exercise in participants had not changed from exercise levels in April (99). Our results support their findings, as PA levels in our study were also low during both lockdown periods in spring and winter. Wijngaards et al. (72) observed a decrease in exercise frequency between April 2020 and January 2021 and subsequently an increase from January 2021 to July 2021 in a representative sample of US adults. It should be noted, however, that in contrast to Europe, different COVID-19 measures were introduced in the US at different times and that sport is also structured and organized differently there, making comparisons between both studies difficult to make.

To our knowledge, no other studies have yet investigated the influence of the severity of COVID-19 measures on PA behavior in similar regions. Tison et al. (70) reported a wide variation in changes in average step count in different countries during the pandemic. While the change in the number of daily steps during the COVID-19 crisis was very similar in several US cities, there were significant differences in the number of daily steps of respondents from different countries (70). Tison et al. (70) also mentioned the lack of comparability between regions as they have different abilities to engage in or gain access to PA. Bosa et al. (100) noted that northern and southern Italian regions differ in terms of their healthcare systems and the extent to which people were affected by COVID-19 and therefore examined different responses of Italian people in terms of COVID-19 crisis management. In the study by Eichenberg et al. (81), emotional stress and well-being of the population in Austria, Germany and Italy were investigated in relation to the severity of the pandemic and emergency measures. Italian respondents had greater concerns about their health and a stronger sense of fear and stress in the early phase of COVID-19 than German or Austrian people (81). These findings are consistent with our results as the individuals participating in our survey from Italy (South Tyrol, Trentino) were also worst affected by the pandemic and had the greatest decline in PA during the COVID-19 crisis.

Our study showed positive and negative associations between different variables and the change in PA over the COVID-19 crisis in 2020. This is in line with the existing literature. Herbec et al. (61) also reported lower probabilities for decreasing moderate to vigorous PA for people with access to a garden/balcony. Due to the temporary closure of public parks and green spaces, domestic gardens become more important. Gardens not only provide opportunities for physical exercise and gardening. Spending time outside on a balcony or in a garden also has a positive influence on well-being (101, 102). As in our study, Moura et al. (103) observed that individuals in Brazil who worked from home during the pandemic were less likely to be physically inactive. Benefits of working from home include shorter travel times to work and more leisure time which can also be used for PA (104). The findings in our study and in the study by Constandt et al. (58), that less time during lockdown led to a drop in exercise, additionally support the previous suggestion. Furthermore, our study showed a greater likelihood of a reduction in PA during the crisis among men than among women. Smith et al. (55) came to the same result, while Qin et al. (42) found greater physical inactivity in women. Maugeri et al. (48) identified a higher significant variation in men compared to women pre and during COVID-19. General gender differences even before the pandemic could be an explanation. Women are more physically active inside the home and do more housework whereas men exercise more frequently outside the home, preferring fitness clubs and PA for competitive reasons (48, 105). For this reason, during the pandemic men were even more restricted in their normal PA behavior than women. In line with previous research (63, 64), we found that participants with psychological distress were more likely to steer away from PA. Feeling more lonely, depressed and anxious during lockdown due to social isolation, uncertainty and unpredictability is a common response to a pandemic (53, 106, 107). In addition to mental health, we observed that a decrease in physical health can also be a predictor for a reduction in PA. In the study by Rogers et al. (63), obesity, hypertension, lung disease and/or a disability to perform activities relating to daily life, were associated with less intensive PA during lockdown.

To explain the change in PA behavior during COVID-19, we used the COM-B system, the center of the behavioral change wheel, of Michie et al. (108). In this system, the behavior results from three components, capability (psychological and physical), motivation (reflective and automatic) and opportunity (physical and social), whereas the behavior also influences the components (108). The COVID-19 crisis may have a negative or positive impact on the components and consequently on the behavior (9). During the pandemic, there were fewer opportunities to be physically active because sports participation was regulated and sports facilities were closed. Fixed training times at sports clubs, regular training at fitness centers or regular sports activities with friends, could no longer take place. This had an impact on motivation. Individuals had to motivate themselves to replace their original PA with other allowed activities. Some people participated in online courses, exercised via apps, purchased sports equipment, did new types of sports or did home workouts (65, 73, 109) as observed in our study. For a large proportion, however, the limited opportunities had a negative impact on motivation and physical activity was reduced during the lockdowns. In our study, we could see that in Trentino which had the strictest lockdown, more people stated that they had found it difficult to stay physically active during the pandemic compared to people in regions with more lenient lockdowns. The main reasons (opportunities, motivation, time) given by our participants are consistent with the components in the COM-B system of Michie et al. (108). Andersson et al. (37) also mentioned that people with an extrinsic PA motivation, who engage in PA for competitive reasons or to socialize while exercising, were more negatively influenced by the crisis than individuals with an intrinsic PA motivation, who want to improve their health and sport-specific skills through PA. Furthermore, we observed that fewer people increased their PA during T4, the second lockdown in November and December 2020, than during the first lockdown in spring 2020. The literature shows that people also reduced their health engagement and their compliance with restrictions as the pandemic progressed (82, 83). For this reason, we suspect that it will become even more difficult for individuals to motivate themselves to exercise and keep fit with each new lockdown with PA restrictions. Looking at long-term results from other crises, similar trends can be found. Okazaki et al. (110) observed a decrease in PA levels in children and adolescents three years after surviving the earthquake and tsunami in Japan 2011. Mattei et al. (111) assessed the impact of the economic crisis in the late 2000s on health-related behaviors in Italy and found an increase in smoking and being overweight between 2000 and 2015. Analysis comparing the period (2006) before the economic crisis in Spain with the period during the crisis (2011–2012) showed a significant negative impact of unemployment on self-assessed health and mental health in the Spanish working-age population (112). In addition, it should be mentioned that PA throughout the year varies with seasonality and weather (113, 114). In warm summer months, sports participation is higher than in colder months (114). In winter when there is more snow, sports behavior also increases (113). This also partly explains the high PA level in summer among participants from the five Alpine regions, the lower PA in spring and fall, and the change in type of sports and locations of PA over time.

The present study has a number of strengths, but is also limited by the following aspects. Highly physically active, healthy and highly educated people were strongly overrepresented in the sample. Despite a higher sports participation in people living in the Alps (39), the percentage of physically active, healthy and highly educated people in the general population of the five regions in the three countries is lower (96, 115, 116). Nevertheless, the informative value of the present study is given because of the large sample size with approximately 3,000 participants. To analyze the PA behavior, we used questions from the Eurobarometer 472 study. The international physical activity questionnaire (IPAQ), a scientifically validated questionnaire, is the most widely used PA questionnaire and provides more detailed information about PA, especially about intensity and sitting time (117). Since we collected the PA data retrospectively for four time points in 2020, the IPAQ would not have provided reliable data because it is unlikely that individuals would remember their detailed PA retrospectively. Due to the underlying ethical guidelines, participants were not required to answer all questions and could leave some questions unanswered. However, this also resulted in incomplete data sets, subsequent data cleaning and ultimately data loss. Before the questionnaire was published, a pretest was conducted to ensure comprehensibility and technical implementation of the questionnaire programming. Nevertheless, it could not be avoided that some participants omitted their answers to the question “amount of PA.” Several sociodemographic variables and lifestyle behaviors were collected to predict changes in PA behavior. Since the adjusted R^2^ for the overall model was 0.18, 18% of the variance of the change in PA over time could be explained by the selected variables. Therefore, there are many other factors that we did not collect or include in the analysis that may have had an impact on the change in PA. In addition, it must be considered, that a reporting bias may exist when using self-reported questionnaires (118).

## Conclusion

To our knowledge, this is the first study to investigate the long-term change in PA levels due to different restrictions during the COVID-19 pandemic in similar Alpine regions. First, the COVID-19 measures, especially stricter regulations, had a negative impact on PA behavior. Since physical inactivity was already a global health issue before COVID-19 (24, 25), the pandemic may have worsened this situation. If the pandemic continues for a prolonged period and people have to endure multiple lockdowns, there will likely be a long-term impact on society and the population's health. For this reason, we recommend that governments always allow moderate PA during pandemics under existing regulations, especially outdoors, which positively influences people's mental and physical health. In addition, parallel to restrictions, governments and other sports providers should develop and implement public health strategies to promote PA and reduce sitting behavior (36, 67). Second, the pandemic also had positive effects on PA behavior, as some respondents increased their PA behavior during the lockdowns. During the crisis, people used apps, online tools or courses, e-sports, changed to home training and bought equipment to remain physically active (65, 119). For sports providers such as sports clubs, fitness centers and schools, the pandemic is a good opportunity to offer sports in a different and digitized way (120). Finally, more longitudinal studies are needed that examine changes in PA and the impact of COVID-19 restrictions on PA throughout multiple lockdowns.

## Data Availability Statement

The data that support the findings of this study are available from the authors upon reasonable request. Requests to access the datasets should be directed to Stefanie.Schoettl@uibk.ac.at.

## Ethics Statement

The studies involving human participants were reviewed and approved by the Institutional Review Board (IRB) of the Department of Sport Science as well as the Board for Ethical Issues (BfEI) of the University of Innsbruck. Written informed consent for participation was not required for this study in accordance with the national legislation and the institutional requirements.

## Author Contributions

SS: project administration, supervision, formal analysis, and writing—original draft. SS and MS: conceptualization, funding acquisition, and methodology. MS, LS, and MK: resources, validation, and writing—review and editing. SS, MS, LS, and MK: investigation. SS, MS, and LS: visualization. All authors contributed to the article and approved the submitted version.

## Funding

The work was supported by funds from Förderkreis der Universität Innsbruck 1669—Wissenschafft Gesellschaft (project number: 328 973). The funding body was not involved in the planning of the study, the analysis and interpretation of data, in writing the manuscript, or the decision to submit the article for publication.

## Conflict of Interest

The authors declare that the research was conducted in the absence of any commercial or financial relationships that could be construed as a potential conflict of interest.

## Publisher's Note

All claims expressed in this article are solely those of the authors and do not necessarily represent those of their affiliated organizations, or those of the publisher, the editors and the reviewers. Any product that may be evaluated in this article, or claim that may be made by its manufacturer, is not guaranteed or endorsed by the publisher.
