# Supplementary material for: Physical Activity Behavior During and After COVID-19 Stay-at-Home Orders—A Longitudinal Study in the Austrian, German, and Italian Alps
Source: Front Public Health. 2022 May 31;10:901763. doi: 10.3389/fpubh.2022.901763 (PMC9194442; doi:10.3389/fpubh.2022.901763)
Supplement: Supplementary file 1 [file Table_1.DOCX]

Supplementary Material

# Supplementary Tables

## Supplementary Table 1

**Supplementary Table 1**: Frequency of PA (never, seldom, 1-3 times a month, 1-2 times a week, 3-4 times a week, 5 times a week or more) over four time points (T1-T4) in five Alpine regions (x² tests were conducted to evaluate significance of differences between frequency of PA and five regions) (PA less than once a week: never, seldom, 1-3 times a month; at least once a week: 1-2 times a week, 3-4 times a week, 5 times a week or more)

| Frequency of PA N (%) |  | T1 | T2 | T3 | T4 |
| --- | --- | --- | --- | --- | --- |
| Tyrol  (n=582) | Never  Seldom  1-3 times a month  1-2 times a week  3-4 times a week  5 times a week or more  Less than once a week  At least once a week | 8 (1.4)  11 (1.9)  38 (6.5)  160 (27.5)  257 (44.2)  108 (18.6)  57 (9.8)  525 (90.2) | 58 (10.0)  51 (8.8)  48 (8.2)  161 (27.7)  146 (25.1)  118 (20.3)  157 (27.0)  425 (73.0) | 11 (1.9)  21 (3.6)  38 (6.5)  150 (25.8)  248 (42.6)  114 (19.6)  70 (12.0)  512 (88.0) | 51 (8.8)  48 (8.2)  62 (10.7)  162 (27.8)  151 (25.9)  108 (18.6)  161 (27.7)  421 (72.3) |
| Vorarlberg  (n=668) | Never  Seldom  1-3 times a month  1-2 times a week  3-4 times a week  5 times a week or more  Less than once a week  At least once a week | 14 (2.1)  21 (3.1)  58 (8.7)  240 (35.9)  232 (34.7)  103 (15.4)  93 (13.9)  575 (86.1) | 56 (8.4)  50 (7.5)  69 (10.3)  171 (25.6)  195 (29.2)  127 (19.0)  175 (26.2)  493 (73.8) | 20 (3.0)  28 (4.2)  46 (6.9)  196 (29.3)  261 (39.1)  117 (17.5)  94 (14.1)  574 (85.9) | 66 (9.9)  58 (8.7)  76 (11.4)  208 (31.1)  164 (24.6)  96 (14.4)  200 (29.9)  468 (70.1) |
| Upper Bavaria  (n=446) | Never  Seldom  1-3 times a month  1-2 times a week  3-4 times a week  5 times a week or more  Less than once a week  At least once a week | 10 (2.2)  15 (3.4)  31 (7.0)  134 (30.0)  179 (40.1)  77 (17.3)  56 (12.6)  390 (87.4) | 33 (7.4)  38 (8.5)  37 (8.3)  117 (26.2)  129 (28.9)  92 (20.6)  108 (24.2)  338 (75.8) | 13 (2.9)  21 (4.7)  22 (4.9)  130 (29.1)  165 (37.0)  95 (21.3)  56 (12.6)  390 (87.4) | 40 (9.0)  52 (11.7)  44 (9.9)  133 (29.8)  106 (23.8)  71 (15.9)  136 (30.5)  310 (69.5) |
| South Tyrol  (n=554) | Never  Seldom  1-3 times a month  1-2 times a week  3-4 times a week  5 times a week or more  Less than once a week  At least once a week | 18 (3.2)  21 (3.8)  44 (7.9)  156 (28.2)  231 (41.7)  84 (15.2)  83 (15.0)  471 (85.0) | 59 (10.6)  66 (11.9)  53 (9.6)  133 (24.0)  140 (25.3)  103 (18.6)  178 (32.1)  376 (67.9) | 19 (3.4)  28 (5.1)  40 (7.2)  150 (27.1)  228 (41.2)  89 (16.1)  87 (15.7)  467 (84.3) | 47 (8.5)  48 (8.7)  51 (9.2)  170 (30.7)  166 (30.0)  72 (13.0)  146 (26.4)  408 (73.6) |
| Trentino  (n=603) | Never  Seldom  1-3 times a month  1-2 times a week  3-4 times a week  5 times a week or more  Less than once a week  At least once a week | 13 (2.2)  20 (3.3)  48 (8.0)  192 (31.8)  233 (38.6)  97 (16.1)  81 (13.4)  522 (86.6) | 122 (20.2)  86 (14.3)  55 (9.1)  145 (24.0)  116 (19.2)  79 (13.1)  263 (43.6)  340 (56.4) | 34 (5.6)  55 (9.1)  48 (8.0)  178 (29.5)  195 (32.3)  93 (15.4)  137 (22.7)  466 (77.3) | 74 (12.3)  82 (13.6)  61 (10.1)  190 (31.5)  135 (22.4)  61 (10.1)  217 (36.0)  386 (64.0) |
| Total  (n=2853) | Never  Seldom  1-3 times a month  1-2 times a week  3-4 times a week  5 times a week or more  Less than once a week  At least once a week | 63 (2.2)  88 (3.1)  219 (7.7)  882 (30.9)  1132 (39.7)  469 (16.4)  370 (13.0)  2483 (87.0) | 328 (11.5)  291 (10.2)  262 (9.2)  727 (25.5)  726 (25.4)  519 (18.2)  881 (30.9)  1972 (69.1) | 97 (3.4)  153 (5.4)  194 (6.8)  804 (28.2)  1097 (38.5)  508 (17.8)  444 (15.6)  2409 (84.4) | 278 (9.7)  288 (10.1)  294 (10.3)  863 (30.2)  722 (25.3)  408 (14.3)  860 (30.1)  1993 (69.9) |
| p-value |  | p=0.061 | p<0.001 | p<0.001 | p=0.001 |

## Supplementary Table 2

Supplementary Table 2: where (**a**) and with whom participants engaged in PA over four time points (T1-T4) (**b**) and in which type of sports they took part (**c**). (Multiple answers were possible)

**a)**

| N (%) |  | T1 | T2 | T3 | T4 |
| --- | --- | --- | --- | --- | --- |
| Tyrol  (n=554) | In a park/outdoors etc.  At home  At a sport club  At a health/fitness center  On the way between home and school/work/shops | 407 (73.5)  136 (24.5)  206 (37.2)  111 (20.0)  51 (9.2) | 339 (61.2)  339 (61.2)  17 (3.1)  1 (0.2)  36 (6.5) | 439 (79.2)  199 (35.9)  149 (26.9)  84 (15.2)  47 (8.5) | 401 (72.4)  299 (54.0)  23 (4.2)  1 (0.2)  37 (6.7) |
| Vorarlberg  (n=632) | In a park/outdoors etc.  At home  At a sport club  At a health/fitness center  On the way between home and school/work/shops | 492 (77.8)  152 (24.1)  178 (28.2)  147 (23.3)  92 (14.6) | 521 (82.4)  256 (40.5)  8 (1.3)  4 (0.6)  69 (10.9) | 523 (82.8)  182 (28.8)  130 (20.6)  102 (16.1)  104 (16.5) | 510 (80.7)  267 (42.2)  8 (1.3)  6 (0.9)  67 (10.6) |
| Upper Bavaria  (n=422) | In a park/outdoors etc.  At home  At a sport club  At a health/fitness center  On the way between home and school/work/shops | 320 (75.8)  108 (25.6)  116 (27.5)  102 (24.2)  56 (13.3) | 342 (81.0)  192 (45.5)  8 (1.9)  6 (1.4)  35 (8.3) | 351 (83.2)  136 (32.2)  81 (19.2)  66 (15.6)  52 (12.3) | 319 (75.6)  212 (50.2)  9 (2.1)  2 (0.5)  32 (7.6) |
| South Tyrol  (n=517) | In a park/outdoors etc.  At home  At a sport club  At a health/fitness center  On the way between home and school/work/shops | 391 (75.6)  121 (23.4)  133 (25.7)  84 (16.2)  50 (9.7) | 279 (54.0)  345 (66.7)  10 (1.9)  0 (0.0)  42 (8.1) | 430 (83.2)  173 (33.5)  77 (14.9)  44 (8.5)  43 (8.3) | 406 (78.5)  251 (48.5)  23 (4.4)  3 (0.6)  33 (6.4) |
| Trentino  (n=573) | In a park/outdoors etc.  At home  At a sport club  At a health/fitness center  On the way between home and school/work/shops | 377 (65.8)  104 (18.2)  174 (30.4)  88 (15.4)  28 (4.9) | 193 (33.7)  392 (68.4)  9 (1.6)  1 (0.2)  26 (4.5) | 427 (74.5)  148 (25.8)  112 (19.5)  34 (5.9)  27 (4.7) | 348 (60.7)  235 (41.0)  56 (9.8)  7 (1.2)  29 (5.1) |
| Total  (n=2698) | In a park/outdoors etc.  At home  At a sport club  At a health/fitness center  On the way between home and school/work/shops | 1987 (73.6)  621 (23.0)  807 (29.9)  532 (19.7)  277 (10.3) | 1674 (62.0)  1524 (56.5)  52 (1.9)  12 (0.4)  208 (7.7) | 2170 (80.4)  838 (31.1)  549 (20.3)  330 (12.2)  273 (10.1) | 1984 (73.5)  1264 (46.8)  119 (4.4)  19 (0.7)  198 (7.3) |

**b)**

| N (%) |  | T1 | T2 | T3 | T4 |
| --- | --- | --- | --- | --- | --- |
| Tyrol  (n=554) | Alone  With family  With friends  In a training group  Under guidance/trainer  With videos/online tools/TV | 286 (51.6)  215 (38.8)  301 (54.3)  203 (36.6)  118 (21.3)  23 (4.2) | 400 (72.2)  211 (38.1)  55 (9.9)  7 (1.3)  14 (2.5)  106 (19.1) | 314 (56.7)  250 (45.1)  291 (52.5)  158 (28.5)  88 (15.9)  56 (10.1) | 390 (70.4)  214 (38.6)  104 (18.8)  23 (4.2)  23 (4.2)  91 (16.4) |
| Vorarlberg  (n=632) | Alone  With family  With friends  In a training group  Under guidance/trainer  With videos/online tools/TV | 346 (54.7)  310 (49.1)  312 (49.4)  190 (30.1)  108 (17.1)  25 (4.0) | 418 (66.1)  341 (54.0)  58 (9.2)  9 (1.4)  18 (2.8)  69 (10.9) | 370 (58.5)  360 (57.0)  283 (44.8)  123 (19.5)  79 (12.5)  40 (6.3) | 416 (65.8)  331 (52.4)  83 (13.1)  9 (1.4)  14 (2.2)  84 (13.3) |
| Upper Bavaria  (n=422) | Alone  With family  With friends  In a training group  Under guidance/trainer  With videos/online tools/TV | 239 (56.6)  178 (42.2)  201 (47.6)  121 (28.7)  79 (18.7)  19 (4.5) | 290 (68.7)  185 (43.8)  62 (14.7)  10 (2.4)  13 (3.1)  72 (17.1) | 249 (59.0)  196 (46.4)  199 (47.2)  86 (20.4)  54 (12.8)  33 (7.8) | 298 (70.6)  172 (40.8)  76 (18.0)  12 (2.8)  13 (3.1)  76 (18.0) |
| South Tyrol  (n=517) | Alone  With family  With friends  In a training group  Under guidance/trainer  With videos/online tools/TV | 271 (52.4)  215 (41.6)  248 (48.0)  142 (27.5)  79 (15.3)  23 (4.4) | 364 (70.4)  191 (36.9)  33 (6.4)  11 (2.1)  16 (3.1)  86 (16.6) | 305 (59.0)  252 (48.7)  234 (45.3)  83 (16.1)  51 (9.9)  38 (7.4) | 354 (68.5)  227 (43.9)  107 (20.7)  16 (3.1)  23 (4.4)  70 (13.5) |
| Trentino  (n=573) | Alone  With family  With friends  In a training group  Under guidance/trainer  With videos/online tools/TV | 288 (50.3)  172 (30.0)  246 (42.9)  175 (30.5)  107 (18.7)  12 (2.1) | 387 (67.5)  161 (28.1)  24 (4.2)  20 (3.5)  10 (1.7)  97 (16.9) | 315 (55.0)  203 (35.4)  225 (39.3)  106 (18.5)  54 (9.4)  35 (6.1) | 354 (61.8)  181 (31.6)  125 (21.8)  62 (10.8)  35 (6.1)  64 (11.2) |
| Total  (n=2698) | Alone  With family  With friends  In a training group  Under guidance/trainer  With videos/online tools/TV | 1430 (53.0)  1090 (40.4)  1308 (48.5)  831 (30.8)  491 (18.2)  102 (3.8) | 1859 (68.9)  1089 (40.4)  232 (8.6)  57 (2.1)  71 (2.6)  430 (15.9) | 1553 (57.6)  1261 (46.7)  1232 (45.7)  556 (20.6)  326 (12.1)  202 (7.5) | 1812 (67.2)  1125 (41.7)  495 (18.3)  122 (4.5)  108 (4.0)  385 (14.3) |

**c)** Participants indicated the 3 most frequently practiced types of sports during the four periods. The types of sports were added together and categorized. Only the most frequently mentioned types of sports can be seen in the Table 2c.

| N (%) |  | T1 | T2 | T3 | T4 |
| --- | --- | --- | --- | --- | --- |
| Tyrol | Mountaineering/hiking  Running/jogging  Cycling  Workout  Walking  Skiing/ski tour  Ball sports/tennis  Swimming  Yoga/pilates/qigong etc. | 132 (11.6)  153 (13.4)  106 (9.3)  116 (10.2)  35 (3.1)  35 (3.1)  85 (7.4)  71 (6.2)  35 (3.1) | 111 (13.7)  150 (18.5)  75 (9.3)  173 (21.4)  75 (9.3)  51 (6.3)  17 (2.1)  18 (2.2)  51 (6.3) | 190 (18.3)  141 (13.6)  163 (15.7)  114 (11.0)  39 (3.8)  33 (3.2)  71 (6.8)  71 (6.8)  33 (3.2) | 139 (16.3)  153 (17.9)  68 (8.0)  153 (17.9)  59 (6.9)  39 (4.6)  17 (2.0)  22 (2.6)  39 (4.6) |
| Vorarlberg | Mountaineering/hiking  Running/jogging  Cycling  Workout  Walking  Skiing/ski tour  Ball sports/tennis  Swimming  Yoga/pilates/qigong etc. | 223 (15.7)  250 (17.6)  190 (13.4)  105 (7.4)  80 (5.6)  113 (8.0)  85 (6.0)  66 (4.7)  64 (4.5) | 229 (20.3)  261 (23.1)  201 (17.8)  120 (10.6)  103 (9.1)  18 (1.6)  23 (2.0)  31 (2.7)  54 (4.8) | 261 (19.5)  241 (18.0)  247 (18.5)  99 (7.4)  75 (5.6)  4 (0.3)  74 (5.5)  104 (7.8)  54 (4.0) | 217 (20.1)  254 (23.6)  118 (10.9)  123 (11.4)  89 (8.3)  62 (5.8)  18 (1.7)  26 (2.4)  62 (5.8) |
| Upper Bavaria | Mountaineering/hiking  Running/jogging  Cycling  Workout  Walking  Skiing/ski tour  Ball sports/tennis  Swimming  Yoga/pilates/qigong etc. | 143 (17.2)  110 (13.2)  120 (14.4)  75 (9.0)  62 (7.4)  58 (7.0)  37 (4.4)  31 (3.7)  33 (4.0) | 136 (19.7)  115 (16.6)  132 (19.1)  92 (13.3)  79 (11.4)  12 (1.7)  12 (1.7)  10 (1.4)  29 (4.2) | 157 (19.7)  95 (11.9)  154 (19.3)  88 (11.1)  64 (8.0)  6 (0.8)  32 (4.0)  43 (5.4)  33 (4.1) | 132 (20.4)  107 (16.5)  73 (11.3)  90 (13.9)  83 (12.8)  25 (3.9)  11 (1.7)  9 (1.4)  40 (6.2) |
| South Tyrol | Mountaineering/hiking  Running/jogging  Cycling  Workout  Walking  Skiing/ski tour  Ball sports/tennis  Swimming  Yoga/pilates/qigong etc. | 175 (17.5)  153 (15.3)  114 (11.4)  99 (9.9)  66 (6.6)  121 (12.1)  57 (5.7)  37 (3.7)  35 (3.5) | 113 (15.3)  137 (18.5)  65 (8.8)  158 (21.4)  77 (10.4)  11 (1.5)  23 (3.1)  11 (1.5)  34 (4.6) | 235 (26.0)  145 (16.0)  162 (17.9)  92 (10.2)  65 (7.2)  5 (0.6)  48 (5.3)  45 (5.0)  25 (2.8) | 155 (20.0)  143 (18.5)  70 (9.0)  117 (15.1)  70 (9.0)  66 (8.5)  19 (2.5)  15 (1.9)  30 (3.9) |
| Trentino  (n=573) | Mountaineering/hiking  Running/jogging  Cycling  Workout  Walking  Skiing/ski tour  Ball sports/tennis  Swimming  Yoga/pilates/qigong etc. | 83 (9.3)  137 (15.3)  109 (12.2)  39 (4.4)  94 (10.5)  82 (9.2)  89 (9.9)  53 (5.9)  19 (2.1) | 53 (8.1)  101 (15.4)  69 (10.6)  95 (14.5)  77 (11.8)  24 (3.7)  37 (5.7)  21 (3.2)  22 (3.4) | 110 (14.4)  133 (17.4)  125 (16.3)  38 (5.0)  97 (12.7)  12 (1.6)  71 (9.3)  36 (4.7)  14 (1.8) | 87 (12.6)  118 (17.1)  87 (12.6)  56 (8.1)  90 (13.1)  49 (7.1)  46 (6.7)  16 (2.3)  18 (2.6) |
| Total | Mountaineering/hiking  Running/jogging  Cycling  Workout  Walking  Skiing/ski tour  Ball sports/tennis  Swimming  Yoga/pilates/qigong etc. | 756 (14.3)  803 (15.2)  639 (12.1)  434 (8.2)  337 (6.4)  518 (9.8)  353 (6.7)  258 (4.9)  186 (3.5) | 642 (16.0)  764 (19.0)  542 (13.5)  638 (15.9)  411 (10.2)  86 (2.1)  112 (2.8)  91 (2.3)  190 (4.7) | 953 (19.7)  755 (15.6)  851 (17.6)  431 (8.9)  340 (7.0)  40 (0.8)  296 (6.1)  299 (6.2)  159 (3.3) | 730 (18.1)  775 (19.2)  416 (10.3)  539 (13.3)  391 (9.7)  266 (6.6)  111 (2.7)  88 (2.2)  189 (4.7) |
